# Single molecule quantitation and sequencing of rare translocations using microfluidic nested digital PCR

**DOI:** 10.1093/nar/gkt613

**Published:** 2013-07-19

**Authors:** Joe Shuga, Yong Zeng, Richard Novak, Qing Lan, Xiaojiang Tang, Nathaniel Rothman, Roel Vermeulen, Laiyu Li, Alan Hubbard, Luoping Zhang, Richard A. Mathies, Martyn T. Smith

**Affiliations:** ^1^School of Public Health, University of California, Berkeley, CA 94720, USA, ^2^Department of Chemistry, University of California, Berkeley, CA 94720, USA, ^3^Department of Chemistry, University of Kansas, Lawrence, KS 66045, USA, ^4^UC San Francisco/UC Berkeley Graduate Program in Bioengineering, University of California, Berkeley, CA 94720, USA, ^5^Division of Cancer Epidemiology and Genetics, National Cancer Institute, NIH, Department of Health and Human Services, Bethesda, MD 20852, USA, ^6^Guangdong Poison Control Center, Guangzhou 510300, China and ^7^Environmental Epidemiology Division, Institute for Risk Assessment Sciences, Utrecht University, Utrecht, NL-3508, The Netherlands

## Abstract

Cancers are heterogeneous and genetically unstable. New methods are needed that provide the sensitivity and specificity to query single cells at the genetic loci that drive cancer progression, thereby enabling researchers to study the progression of individual tumors. Here, we report the development and application of a bead-based hemi-nested microfluidic droplet digital PCR (dPCR) technology to achieve ‘quantitative’ measurement and single-molecule sequencing of somatically acquired carcinogenic translocations at extremely low levels (<10^−6^) in healthy subjects. We use this technique in our healthy study population to determine the overall concentration of the t(14;18) translocation, which is strongly associated with follicular lymphoma. The nested dPCR approach improves the detection limit to 1 × 10^−7^ or lower while maintaining the analysis efficiency and specificity. Further, the bead-based dPCR enabled us to isolate and quantify the relative amounts of the various clonal forms of t(14;18) translocation in these subjects, and the single-molecule sensitivity and resolution of dPCR led to the discovery of new clonal forms of t(14;18) that were otherwise masked by the conventional quantitative PCR measurements. In this manner, we created a quantitative map for this carcinogenic mutation in this healthy population and identified the positions on chromosomes 14 and 18 where the vast majority of these t(14;18) events occur.

## INTRODUCTION

Tumor-specific somatic mutations can provide highly useful molecular biomarkers and therapeutic targets for cancer diagnosis, prognosis and treatment. Central to the use of these genetic biomarkers in clinical oncology is sensitive and quantitative measurement of rare mutations in a vast excess of wild-type alleles. For instance, discovering driver mutations that lead to carcinogenesis in a rare subset of cells is one key approach to the risk assessment, early detection and treatment of cancer (1,2). Investigation of genetic variants in rare circulating tumor cells in metastatic cancer patients would help understand the biology of metastasis and development of drug resistance in chemotherapy (3). Moreover, quantification of low-level mutated sequences in cancer patients during and after treatments can provide informative data for evaluating therapy efficacy, monitoring minimal residual diseases and detecting disease relapse (4).

In recent years, technical advances have enormously improved the capacity to analyze genetic variants, yielding novel methods for the detection of rare mutations (5). For instance, quantitative PCR (qPCR), a widely used approach in genetic analysis, measures the analog fluorescence signal of targets and thus is limited in the detection sensitivity and/or quantification accuracy owing to instrumental and experimental variation. An attractive alternative to this analog technique is digital PCR (dPCR), which provides a superior sensitivity to conventional qPCR by allowing absolute quantification of target molecules (6–9). Here, we report the development and application of a bead-based hemi-nested microfluidic digital droplet PCR (simplified as nested dPCR hereafter) approach to achieve ‘quantitative’ measurement of somatically acquired carcinogenic translocations at extremely low levels (<10^−6^) in healthy subjects. This sensitive nested dPCR approach has an overall clinical sensitivity that is mainly limited by the amount of DNA that is available for screening (10). In contrast to other dPCR methods using emulsion droplets (8,9), our bead-based dPCR approach provides not only superior quantification performance at extremely low levels but also the capacity to sequence and quantify each mutated clone in a subject after millions of discrete single molecule reactions are conducted in parallel. Therefore, this novel dPCR method can be used to measure the amounts of various clones within a subject or population over time and thus monitor for clonal expansion before clinical disease progression.

The model translocation that we chose for technology validation, the *BCL-2/immunoglobulin heavy chain (IgH)* translocation t(14;18), is highly prevalent in many blood cancers, including ∼80% of follicular lymphoma (FL) cases and ∼25% of large-cell B-cell lymphoma cases (11,12). The translocation brings the B-cell lymphoma-2 (*BCL2*) gene from 18q21 under the control of the strong enhancers of the *IgH* locus, ultimately disrupting *BCL2*’s normal pattern of expression in B cells (13,14). *BCL2* is an anti-apoptotic protein, and its overexpression can be intimately involved in the pathogenesis of B-cell neoplasms (15). t(14;18) is found in a relatively small fraction of the peripheral blood mononuclear cells (PBMCs) of healthy individuals and may be a biomarker of early lymphoma (16–18). The mutation concentration in healthy individuals is ∼1000-fold lower than for individuals with stage III/IV FL(10), and it is believed that clonal expansion of atypical B cells is required for lymphoma progression (16,18–20). t(14;18) prevalence at any level in healthy populations has been reported in the range of 8–88%, which reflects the differences both in the populations studied and in the techniques used to assay t(14;18) (17,21,22). Thus ‘highly sensitive and quantitative detection’ of t(14;18) is essential for fully investigating the clinical value of t(14;18) for risk assessment and early diagnosis of lymphoma. Furthermore, clinical studies have observed clonal evolution of t(14;18) associated with disease progression in individual patients (23). A high-throughput technique that can sequence and quantify multiple t(14;18)^+^ clones could provide insight into the molecular pathology and clinical importance of t(14;18) (24,25).

Using the nested microfluidic dPCR method, we were able to quantitatively detect and sequence a single t(14;18) copy in 9 µg (∼3 × 10^6^ copies) of clot genomic DNA (gDNA) from individuals in a healthy study population. We also applied nested dPCR to develop a quantitative genomic map of t(14;18) by sequencing and quantifying the unique t(14;18) clones found in individual subjects within this study population. The genomic map that we produced represents a baseline for this healthy population, and further sampling of this population can be used to monitor for expansion of particular clonal forms as part of disease progression.

## MATERIALS AND METHODS

### Study subjects

The formaldehyde-exposed worker population, exposure assessment and biological sampling from the study subjects were described in detail in the Supplementary Methods.

### Cell culture

Cell lines of t(14;18)^−^ TK6 (CRL 8015) and t(14;18)^+^ RL (CRL 2261) (ATCC, Manassas, VA) lymphoblasts were maintained in RPMI 1640 medium (Gibco, Carlsbad, CA) supplemented with 10% fetal bovine serum (FBS) (Omega Scientific, Tarzana, CA) at a cell density of (2 × 10^5^ − 2 × 10^6^)/ml and incubated at 37°C in a 5% CO_2_ atmosphere.

### Cell and gDNA purification

We used Clotspin® baskets and the Gentra® Puregene® blood kit (Qiagen, CA) to purify clot gDNA. Buffy coat was prepared by spinning whole blood at 200*g* for 10 min and then by removing the concentrated leukocyte band. We then isolated DNA using the FlexiGene DNA kit (Qiagen). PBMCs were purified from whole blood using density gradient centrifugation through Ficoll-Paque™ PLUS following the manufacturer’s recommendations (GE Healthcare, NJ). To purify gDNA from cell lines and PBMCs, we used a standard cell lysis with RNA and protein digests followed by a phenol–chloroform DNA extraction. The quality of gDNA was assessed, and copy number was normalized using qPCR for β-actin. For more detail regarding gDNA extraction and quality assessment, please see the Supplementary Methods.

### Microfluidic droplet-based dPCR

The four-channel Microfabricated emulsion generator array (MEGA) devices were constructed and operated as detailed previously (26). For droplet generation, freshly prepared carrier oil was injected into the oil channels by a syringe pump, and droplet dPCR mix containing t(14;18) amplicon and primer-conjugated beads was driven by the on-chip diaphragm pump, which was pneumatically actuated by a solenoid valve controller system built in house. The pumping was conducted in a four-step fashion under the control of a LABVIEW program to produce uniform ∼2.5 nl of PCR droplets, which were collected in 0.5 ml of PCR tubes filled with microfine emulsion. Thermal cycling was carried out in a PTC200 thermocycler (MJ Research) and involved a 10 min hot start at 95°C, and 33 cycles of 95°C for 30 s, 60°C for 60 s, 72°C for 90 s and a final 72°C extension for 5 min. The beads were then recovered by using a 15 μm of mesh filter, rinsed with isopropanol, ethanol and 1× Dulbecco’s PBS (DPBS, GIBCO) and analyzed by a multicolor flow cytometer (FC-500, Beckman-Coulter). More details regarding chip fabrication, droplet generation, PCR condition and bead handling and flow cytometry were provided in the Supplementary Methods.

### Hemi-nested PCR

**Figure 1.**
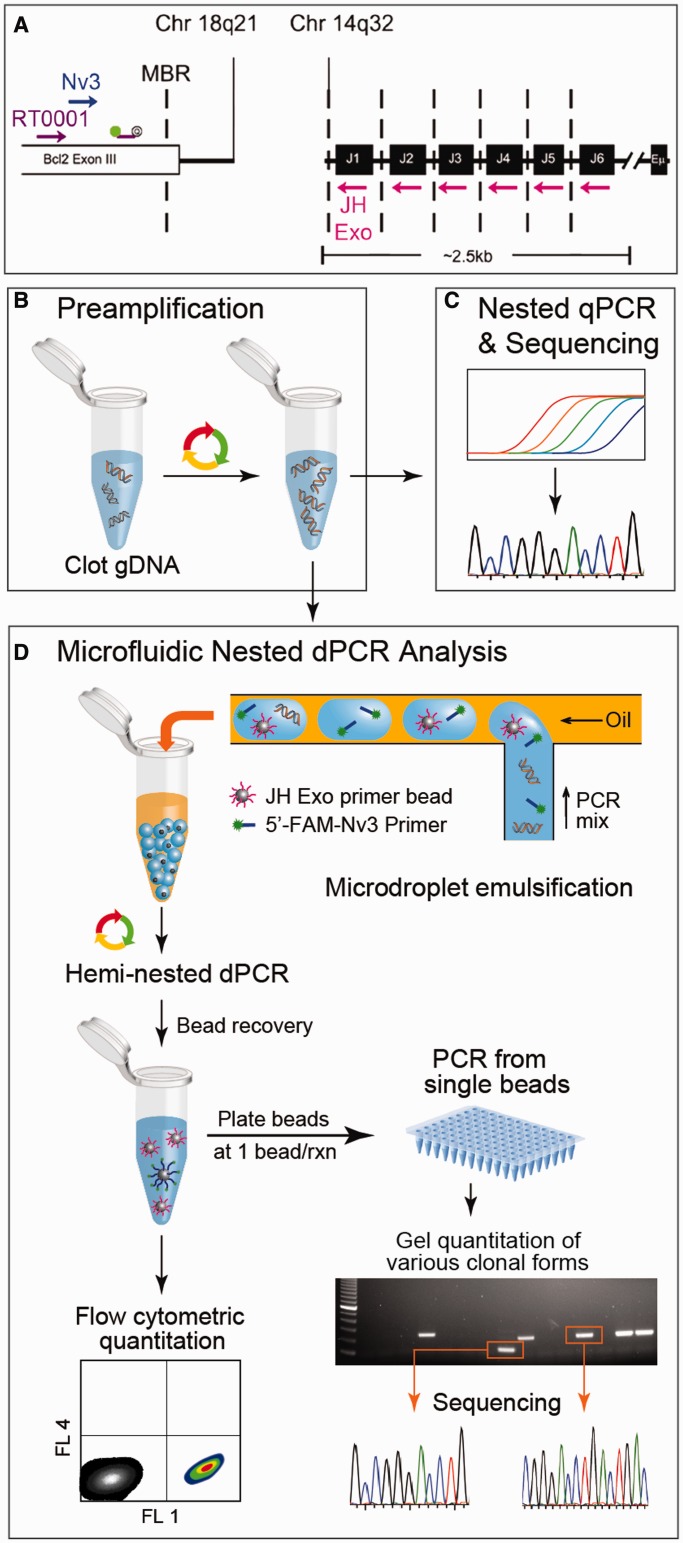
Workflow and methodology for digital detection of t(14;18) in the clot gDNA of healthy subjects. (**A**) The PCR strategy for t(14;18) detection is illustrated by mapping the primers and probes used to their genomic targets. The first round of PCR was primed with the outer primer pair [consisting of an *IgHJ* consensus primer (JH Exo) and a *BCL*2-specific primer (RT0001)]. The hemi-nested secondary rounds of PCR were primed with a nested BCL2 primer (Nv3) while retaining the use of the IgHJ consensus primer (JH Exo). (**B**) A preamp PCR (20 cycles) produced t(14;18) amplicon from 3 µg of clot gDNA. The product of this first amplification step was then used as template in secondary rounds of hemi-nested PCR. Two different types of quantitative PCR were conducted: (**C**) standard qPCR using a BCL2-specific fluorescent probe (see Supplementary Figure S2); and (**D**) microfluidic emulsion single-molecule PCR using JH Exo-functionalized beads and 5′-FAM-labeled Nv3 for t(14;18) detection. A microfluidic emulsion generator array was used for high throughput (>10^6^/h) production of monodisperse reaction volumes. Following emulsion generation, the nanoliter-scale reaction droplets were cycled to achieve single copy genetic analysis of the template. If a copy of t(14;18) and a bead were both present in a reaction droplet, then the bead was labeled with fluorescent amplicon during PCR, otherwise beads remained unlabeled. Beads were then recovered and analyzed by flow cytometry to determine t(14;18) concentration within a sample. In addition, some of the beads were distributed at ∼1 bead/well in 96-well plates to confirm that FAM+ concentration among beads corresponds with t(14;18)+ concentration as determined using a *BCL2*-specific probe. This tertiary round of amplification also produced sufficient template to conduct standard sequencing reactions that were derived from single molecule reactions.

All oligonucleotides (Supplementary Table S3) used in this study were obtained from IDT (Coralville, IA), and, unless otherwise noted, PCR reagents were from Life Technologies (Carlsbad, CA). The pre-amplification (preamp) reaction mix contained 1× *Ampli* Taq Gold® buffer with 5 mM MgCl_2_, 0.2 mM deoxyribonucleotide triphosphates (dNTPs) (deoxyuridine triphosphate (dUTP) was used at 0.4 mM instead of deoxythymidine triphosphate (dTTP)), 0.01 U µl^−1^ of uracil-DNA glycosylase (UDG) (Roche), 2.5% dimethyl sulfoxide (DMSO), 0.3 µM each of the oligonucleotides (JH Exo and RT0001), 0.035 U µl^−1^ of *Ampli* Taq Gold® Polymerase and 3 µg of gDNA per 50 µl reaction. Thermal cycling in an ABI GeneAmp® 9700 cycler consisted of a UDG reaction (50°C for 2 min), followed by a 10 min hot start at 95°C and 20 cycles of 95°C for 15 s, 60°C for 30 s, and 72°C for 30 s. The hemi-nested reaction mix contained the same components as the preamp mix except that 1 µM ROX reference dye, 0.3 µM each of the primers (JH Exo, Nv3, and BCL2MBRTM2) and 1 µl of the preamp reaction product were used instead. Thermal cycling in an ABI 7300 cycler consisted of a 10 min hot start at 95°C, and 33 cycles of 95°C for 15 s, 60°C for 30 s and 72°C for 30 s. Standards made by diluting RL gDNA in TK6 gDNA were used to establish a calibration curve for t(14;18) quantification (Figure 1 and Supplementary Figure S2). Each assay plate included three standards (10^2^, 10^1^ and 3 copies in 3 µg) and a negative control (3 µg of TK6 DNA) to ensure robust and specific detection at the level of 1 copy/µg. Positive reactions were run on a 1.5% agarose gel to separate amplicons that were then excised, purified using a QIAquick Gel Extraction Kit (QIAGEN) and sequenced at the UC Berkeley Core Sequencing Facility.

### Single-molecule sequencing

Amplicon-bound beads from dPCR were counted using a hemocytometer, plated in 96-well PCR plates at ∼1 bead per well, and re-amplified using the hemi-nested PCR conditions described in the last section. DNA amplicons yielded from single beads were sequenced following the method described earlier in the text. Sequencing reads were aligned to the reference assembly of the human genome using NCBI’s nucleotide basic local alignment search tool (BLASTn). The ‘N sequence’ insert was identified as the *de novo* sequence found between the two breakpoints in a particular translocation clone. To map the locations of the V(D)J recombination signal sequences (RSSs), we mapped all allowable RSS nonamers and heptamers to the chromosome 14 contig (NT_026437.11) and then found nonamer–heptamer pairs that were separated by appropriately sized spacer sequences (Figure 5 and Supplementary Figure S6). More details about single-molecule sequencing and sequence analysis were discussed in the Supplementary Methods.

## RESULTS

### Digital quantitation and single-molecule sequencing of t(14;18): method design and performance

We found that our standard qPCR method was not sensitive enough to quantify and sequence t(14;18) from the clot gDNA of healthy subjects (Supplementary Figure S1); therefore, we developed a nested PCR approach (Figure 1a and c) for digital analysis of t(14;18). This approach starts with a preamp reaction (Figure 1b), and the resultant target copies are then quantified and sequenced using both a conventional nested qPCR method and the microfluidic nested dPCR for direct comparison of their performance. The nested qPCR detection was conducted in 50 µl of reaction volumes with a *BCL2*-specific cleavable probe sequence (Figure 1c and d) to determine the threshold cycle (C_t_) values (Figure 1c and Supplementary Figure S2). The dPCR methodology uses our custom-built MEGA devices and a bead-based emulsion PCR assay (26) to achieve high-throughput digital quantitation and single-molecule sequencing of t(14;18) (Figure 1d). In this methodology 2.5 nl of droplets serve as digital reaction volumes and droplets containing both single copies of t(14;18) and an IgH primer-functionalized bead yield clonal DNA beads labeled by fluorescein amidite (FAM)-labeled BCL2 primer after thermal cycling. A portion of the post-PCR beads are then analyzed by flow cytometry to quantify the target copies. Remaining beads are used as templates for further PCR amplification for single-molecule counting and sequencing of the genetic variants of the mutation (Figure 1d).

**Figure 2.**
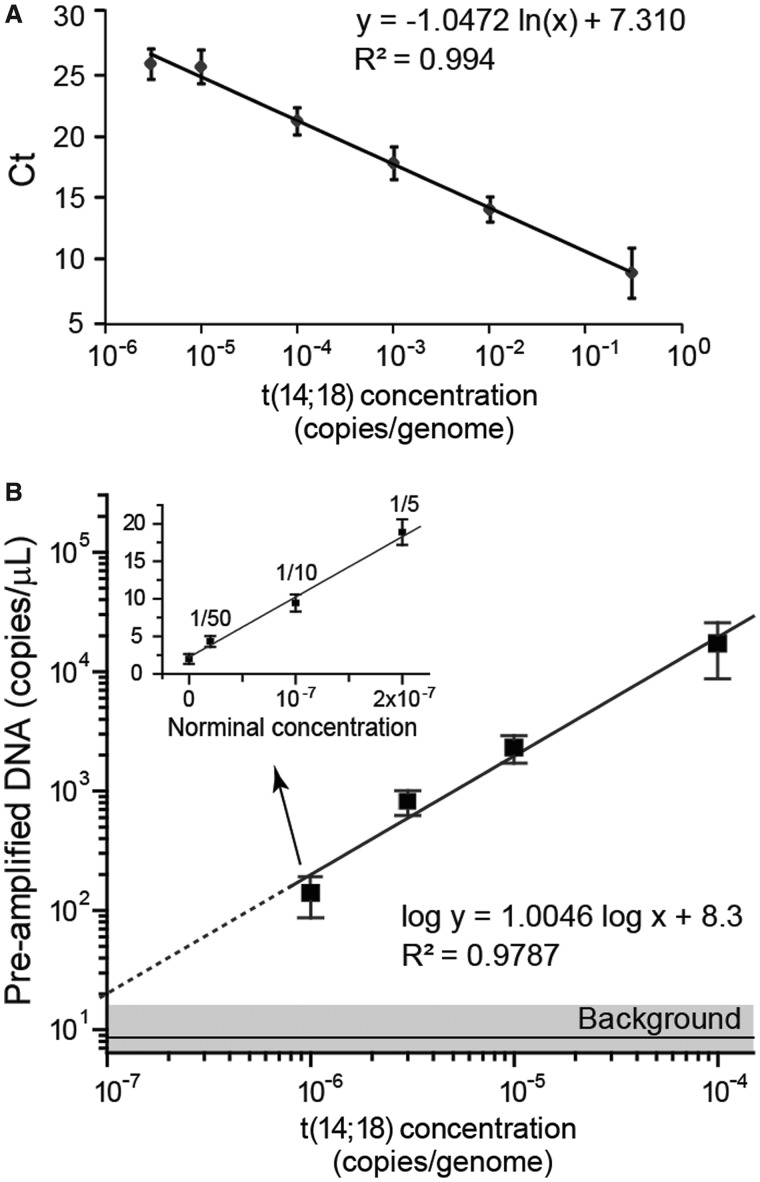
Characterization of hemi-nested real-time PCR (qPCR) and microfluidic dPCR assays for quantitation of the t(14;18) translocation. (**A**) The hemi-nested qPCR analysis of t(14;18) copies spiked in negative human genomic DNA shows a linear standard curve of the threshold cycle (Ct) as a function of the t(14;18) concentration [copies of t(14;18)/total genomic copies] with a dynamic range spanning five orders of magnitude and a limit of detection near 10^−6^ copies t(14;18)/genome. The error bars represent standard deviation (*n* = 3). (**B**) The hemi-nested microfluidic dPCR assay quantitatively measures t(14;18) concentration with a limit of detection on the order of 10^−7^. The error bars represent standard deviation (*n* ≥ 3). The inset demonstrates quantitative measurements of further dilutions (down to 1/50) of the pre-amplified product from the lowest concentration standard [∼10^−6^ copies t(14;18)/genome]. The error bars indicate standard error.

Figure 2 directly compares the detection performance of both nested assays using the standards of t(14;18)^+^ gDNA spiked into wild-type human gDNA. The qPCR methodology had an efficiency of 93.8% and a linear dynamic range that spanned five log_10_ t(14;18) concentrations (Figure 2a). Each 50 µl of reaction had a quantitative limit of ∼3.3 × 10^−6^ copies of t(14;18) per genome (1 copy/µg gDNA) with an ultimate limit of detection of ∼10^−6^ copies of t(14;18) per genome (1 copy in 3 µg of gDNA) (Figure 2a). The microfluidic nested dPCR technique offered quantitative detection down to a concentration of ∼10^−6^ copies t(14;18) per genome (1 copy in 3 µg of gDNA), and the theoretical detection limit was determined to be 10^−7^ copies per genome as the experimental signal is well above the background noise (Figure 2b). Further dilutions of the lowest concentration preamp standard were used to demonstrate quantitative detection down to equivalent concentrations near ∼2 × 10^−8^ (Figure 2b, inset), indicating the detection and quantitation limits of the microfluidic nested dPCR detection is constrained by the amount of DNA input (maximum of 3 µg) that can be used in the 50 µl of preamp reactions.

### Digital detection and quantification of t(14;18) in occupationally exposed subjects

**Figure 3.**
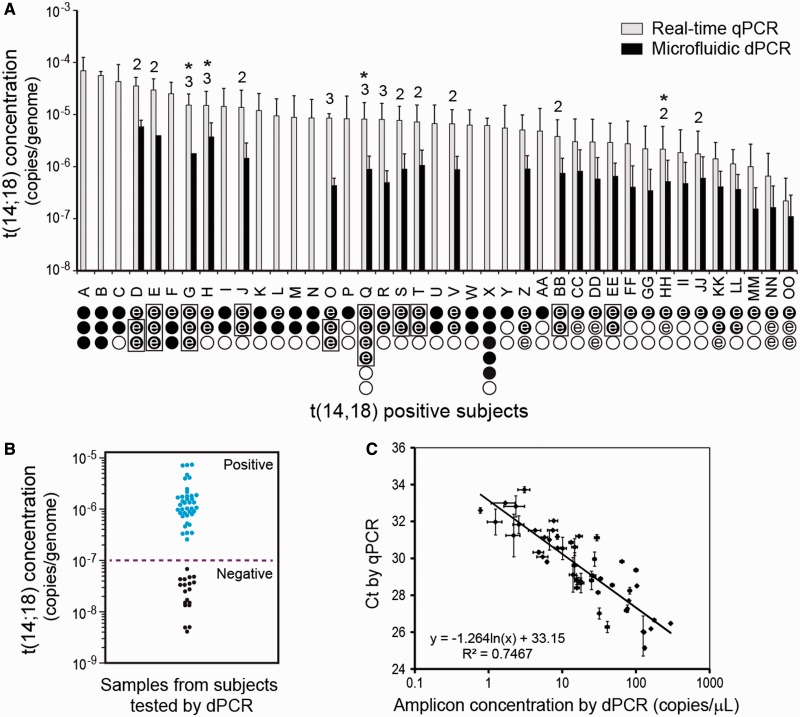
Concentration of t(14;18) in positive study subjects. We detected t(14;18) in the clot gDNA of 41/93 healthy test subjects (∼44% prevalence). (**A**) Subject IDs are given on the abscissa, and the bar-height indicates the average concentration of t(14;18) in a given subject as determined by the hemi-nested PCR techniques. The error bars indicate the standard deviation in the measurement. Below the subject ID on the abscissa, the number of 3 µg of preamp reactions conducted on the subject’s gDNA is indicated by the total number of circles. Filled circles indicate that a preamp reaction was positive by standard qPCR, whereas open circles indicate that the preamp reaction was found negative by standard qPCR. If an assay circle is marked with the letter ‘e’, then that preamp reaction was also tested using microfluidic PCR, and in these cases, the quantitative result from dPCR is also given. A box grouping preamp reactions indicates that these reactions were pooled before dPCR analysis. The data from these parallel measurements (when positive) contributed data to part c of this figure. For all positive subjects, at least one clonal form of t(14;18) was confirmed by sequence analysis; in some subjects, multiple clonal forms of t(14;18) were defined. If multiple clonal forms were found in a subject, the total number of defined clones is indicated above the error bars. An asterisk indicates that one clonal form for that subject went undetected in qPCR and was then discovered and/or defined in dPCR. (**B**) The dot scattered plot of the nested dPCR measurements of the preamp reactions showing the distinct populations for the t(14;18) positive and negative subjects. The dashed line indicates the detection limit of 10^−7^ determined in Figure 2. (**C**) The two methods represented in Figure 1c and d correlate well for quantitating t(14;18) both in cell line-derived DNA standards and in clot DNA samples from chemically exposed workers. The horizontal error bars are standard error and the vertical error bars are standard deviation (*n* = 2).

To validate the microfluidic dPCR method for digital analysis of rare t(14;18) mutations, we examined the clot gDNA samples of 93 healthy Chinese subjects, 42 of whom had been occupationally exposed to formaldehyde. All the samples were first characterized using the bulk nested qPCR method (Figure 1b and c). Three aliquots of 3 µg of gDNA from each subject were pre-amplified and assayed (Figure 3a, circles under each subject indicate number of assays). Two samples from Subjects Q and X are included for internal quality control, giving six reactions for each of these subjects. We found that there were detectable levels of t(14;18) in 41 of 93 (∼44%) study subjects, as summarized in Figure 3a. Thirty-two of these t(14;18)^+^ subjects show one or more negative reactions (indicated by the open circles in Figure 3a), presumably owing to the stochastic distribution of rare targets in the 3 µg of gDNA aliquots. The overall concentration in these t(14;18)^+^ subjects ranged from ∼7.7 × 10^−5^ (∼23 copies/µg) in Subject A down to ∼4 × 10^−7^ (a single copy in a total of 9 µg of gDNA assayed) in Subject OO. The median level of t(14;18) in the 41 positive study subjects was 2.23 copies/µg, and the mean level of t(14;18) among the positive study subjects was 3.83 copies/µg (genomic concentrations of 7.4 × 10^−6^ and 1.3 × 10^−5^, respectively). Furthermore, we used sequencing and sequence analysis to definitively confirm and fully define at least one clonal form in all 41 t(14;18)^+^ study subjects and to confirm that the forms of t(14;18) found in these subjects were unique and different from that found in the positive control CRL 2261 cell line. In several subjects, more than one unique clonal form was identified, and for these subjects, the total number of t(14;18) clones identified is displayed above the bars for each subjects (Figure 3a).

To assess the microfluidic-nested dPCR technology for rare translocation detection and molecular profiling, we focused our studies on the subjects with extremely low concentration of t(14;18) and/or multiple clonal forms. We analyzed a total of 69 preamp reactions, which consisted of 50 positive reactions and 10 negative reactions from 28 t(14;18)^+^ subjects (circles marked with ‘e’ in Figure 3a) and 9 reactions randomly chosen from t(14;18)^−^ subjects. The t(14;18) concentration results obtained by the nested microfluidic dPCR analysis agreed with those obtained by the standard qPCR. This parallel comparison also demonstrated the robust nature of both ultrasensitive assay variants. There was not a single instance of disagreement between the two methods for determination of positive/negative preamp reactions. We assayed 10 negative preamp reactions from seven positive subjects at the low end of t(14;18) concentration as determined by qPCR. All the nested dPCR assays yielded consistently negative results, a finding that strongly suggests true negatives, given the sensitivity of the methods used. Only one of the three preamp reactions from many of the subjects contained t(14;18), and the single positive qPCR trial for some subjects (e.g. subject OO with a C_t_ value of 28.8), corresponded to ∼1 copy in 3 µg of gDNA, which suggests digital t(14;18) detection in the single positive assay reaction for these subjects. These observations demonstrate the ability of these nested methodologies to detect a single copy of t(14;18) in 9 µg of gDNA (relative genomic concentration of ∼4 × 10^−7^). Thus, the overall clinical sensitivity of this method, like other ultrasensitive techniques that achieve mutation detection at the single copy level, is limited mainly by the amount of gDNA available for screening (10,27).

The scatter plot of the nested dPCR measurements of the preamp reactions (Figure 3b) shows that the positive population is distinctly separated from the negatives, which represent an extremely low background (below genomic concentration of 10^−7^). Such detection performance and background level are consistent with those obtained using the gDNA standards. The measured concentration of some positive reactions was lower than the lowest concentration possible in our assays using 3 µg of gDNA (1 × 10^−6^), which we found is largely attributed to the degradation of DNA caused by the freeze-thaw of the preamp samples. Regression of the parallel measurements from the t(14;18)^+^ preamp reactions showed good correlation between the microfluidic nested dPCR and the standard nested qPCR (R^2^ ∼0.75, Figure 3c). Overall, these observations confirm that our nested dPCR methodology is ultrasensitive and capable of quantifying rare mutations at concentrations of 10^−6^ and lower.

In addition, the same clonal form(s) of t(14;18) were identified using both nested techniques for all positive preamp reactions, except that an additional form of t(14;18) was discovered by microfluidic nested dPCR for two subjects (J and Q, note asterisks in Figure 3a). This discovery demonstrates a key advantage of dPCR: single-molecule analysis allows each clonal form to be amplified, detected and quantified without competition from other clonal forms, whereas a high concentration clonal form can mask the presence of a less concentrated clone in bulk analysis. Beyond the discovery of novel low-frequency clones, the dPCR technique can also be used to discretely amplify similarly sized clones that are contained within a single sample, and thus allow these distinct clonal forms to be resolved (see discussion of subjects H and HH in the next ‘Results’ section). The results from single-molecule quantification and sequencing are detailed later in the text.

### Single-molecule sequencing to define and quantify t(14;18) clones

**Figure 4.**
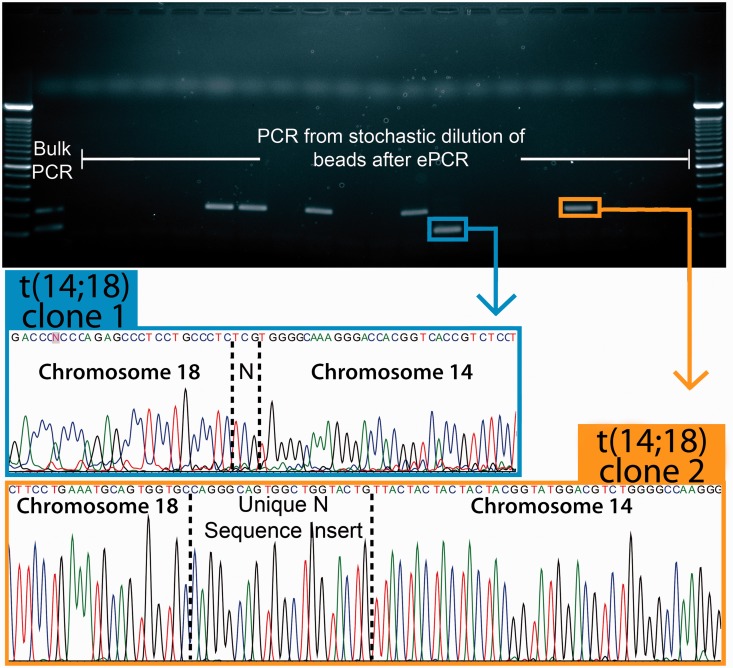
Definition and quantitation of clonal forms within subject samples. Representative results for the gel and sequence analysis of a single subject (‘D’) who was found to be positive for two unique clonal forms of t(14;18). The gel bands were excised for sequencing reactions, and sequence analysis defined the clonal forms of t(14;18) present in the sample. In bulk assays, both clonal forms amplify together, and it is not possible to estimate their relative concentration. However, in digital microfluidic dPCR, the different clonal forms are discretely encapsulated in nanoliter-scale reaction droplets along with primer-functionalized beads. The resulting digital reactions load individual beads with amplicon that represents only a single form of the translocation. By simply counting the number of ‘large’ and ‘small’ bands or sequence reads, the relative ratio of clonal forms within a sample can be estimated. In this gel section, the ratio of clone 1:clone 2 is 1:5, but gel analysis of more reactions from this subject estimates the ratio as ∼18:37 (see Supplementary Figure S3b).

To fully define the various t(14;18) clones and to definitively confirm positive assay reactions, we purified various clonal forms of t(14;18) by size using agarose gel electrophoresis and then extracted the amplicons for sequencing reactions. When multiple clones are present in the same preamp reaction, the standard ‘bulk’ nested qPCR technique yields multiple bands in the same lane, and it is impossible to estimate the relative ratio of clonal forms. However, when the same preamp reaction is analyzed using the microfluidic nested dPCR technique, each positive reaction droplet amplifies a single molecule of t(14;18) amplicon (Figure 4 and Supplementary Figure S3). The gel and sequencing data from single molecule-derived sequencing reactions can then be used to estimate the relative concentration of each clonal form in a preamp reaction. Following the nested dPCR from one particular preamp reaction conducted on subject D, flow cytometry revealed that the resulting primer beads were 27.8% positive for t(14;18) (Supplementary Figure S3a). A representative section (19/96 wells) from a plate of single-molecule sequencing reactions (∼1 bead/reaction on average) is displayed in Figure 4 and reveals close agreement in the concentration of beads that are t(14;18)^+^ [6/19 (∼31.6%) versus 27.8%]. Further, this section of the plate estimates the relative ratio of clone 1: clone 2 as 1:5 (Figure 4). More single-bead reactions from two 96-well plates were considered and provided a better quantitative agreement with cytometry [53/192 (∼27.6%) versus 838/3017 (27.8%)] and a more accurate estimate of the ratio of clone 1: clone 2 as 18:37 (Supplementary Figure S3b). The overall C_t_ from qPCR for this preamp reaction was 22.15, corresponding to ∼18 copies per µg or a genomic concentration of ∼6 × 10^−5^ copies per genome. When the data from both methods are considered, we can estimate that clone 1 is present at a concentration of ∼2 × 10^−5^ copies per genome, and that clone 2 is present at a concentration of ∼4 × 10^−5^ copies per genome. This analysis demonstrates the key strength of single molecule analysis via dPCR: each clonal form can be uniquely quantified and tracked. We used this approach to develop a quantitative map of the t(14;18) landscape in our healthy Chinese study population (see next ‘Results’ section).

Another advantage of dPCR is that the technique can be used to resolve similarly sized clones carried by a subject. Subjects H and HH carried similarly sized clones, and the conventional bulk qPCR approach was unable to resolve the clonal forms present in preamp reactions. In these cases, the various clones of t(14;18) that were concurrent in preamp reactions were similar in size and were not adequately separated on gel before purification for sequencing reactions. Subsequent sequencing reactions yielded reads with consensus near the Nv3 sequencing primer but with mixed traces as the clonal forms diverged (Supplementary Figures S4a and S5a for Subjects H and HH, respectively). Therefore, we used the microfluidic nested dPCR method to discretely package the various clonal forms of t(14;18) before nested amplification, and we then used a tertiary round of PCR to produce sufficient amplicon for sequencing. In both cases, dPCR and single-molecule-derived sequencing allowed us to purify and define the similarly sized clones of t(14;18) that were present in a particular preamp reaction (Supplementary Figures S4b, c and S5b and c).

To assess the effect of sequencing errors, we used a tracking sequence on the BCL2 side of the translocation, which is less variable than the IgHJ side of the translocation. The tracking sequence (AGAGCCCTCCTGCCCT) extends from position 3498 to position 3513 on NM_000633.2, and it was chosen because it appears in the vast majority of sequencing reads owing to both its proximity to the Nv3 sequencing primer and its distance from most of the BCL2 breakpoints. The single exception is that of clone 1 from Subject S; this clonal form [3507-N- 87331509 (BCL2-N-IgHJ)] had a BCL2 breakpoint at 3507 and therefore did not contain the complete tracking sequence. Overall, we observed that 67% of all sequencing reads from bead-based amplifications contained no errors in this tracking sequence, whereas 33% of reads contained at least one error. The most common type of sequencing error were insertions (50% of all observed errors), whereas substitutions accounted for 30% of all errors, and multiple errors accounted for the remaining 20% of misread sequences. However, these types of sequencing errors did not impact the results presented here, as each clonal form of t(14;18) was sequenced multiple times, allowing a definitive consensus sequence to be assembled, despite the presence of sequencing errors.

### Quantitative genomic mapping of t(14;18) breakpoints

**Figure 5.**
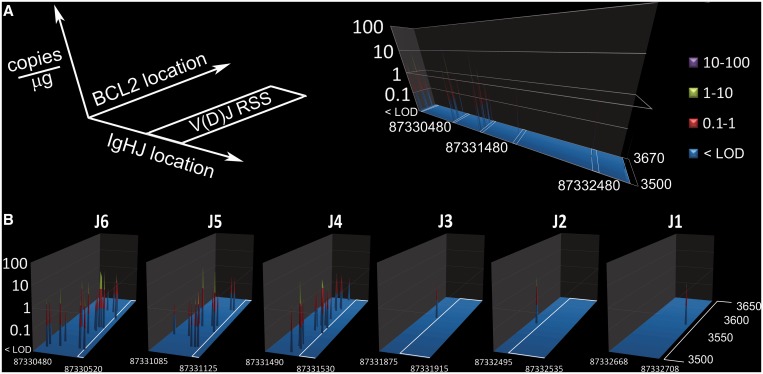
Quantitative genetic profile of t(14;18) breakpoints in positive study subjects. The clonal form of t(14;18) defined in this study are quantitatively mapped to their breakpoint coordinates on Chromosomes 14 (position on NT_026437.11) and Chromosomes 18 (position on NM_000633.2). Most positive subjects (27/41) were positive for a single, unique form of t(14;18); however, 9/41 positive subjects were confirmed to be positive for two unique clonal forms, and 5/41 were confirmed to be positive for three unique clonal forms (also see Figure 3 and Supplementary Table S1). (**A**) The amount and coordinates of these 60 clonal forms of t(14;18) are mapped to a global genetic plot. The white boxes on the plot indicate the locations of the IgHJ RSSs that direct V(D)J recombination. (**B**) A magnified view of each t(14;18) cluster is shown; all clusters were found on the heptamer side of an IgHJ RSS. For details of sequence analysis results on these clonal forms, see Supplementary Table 1. For details regarding the mapping of the IgHJ RSSs to NT_026437.11, see Supplementary Figure S6.

We defined all of the clonal forms present in positive assay reactions and found that most positive subjects carried a single unique t(14;18) clone, although nine subjects carried two unique clonal forms and five subjects carried three unique clonal forms. We used the dPCR method to resolve each t(14;18) clone in subjects carrying multiple clonal forms, and thus we were able to quantify the relative amount of each clonal form. These relative ratios were then scaled by the results from conventional qPCR, which can only measure the total amounts of t(14;18) within a subject, to quantify all 60 clonal forms that were defined in this study. A quantitative genomic map of the chromosome 14 and chromosome 18 breakpoints for the 60 t(14;18) clones identified in this healthy Chinese population shows typical breakpoint clustering along both chromosomes for this translocation (Figure 5).

The chromosome 14 breakpoints were always found on the heptamer side of V(D)J RSSs, with most chromosome 14 breakpoints occurring near the J4, J5 or J6 RSS (Figure 5, Supplementary Figure S6, Supplementary Table S1). This clustering on chromosome 14 is consistent with the theory that errors in V(D)J recombination are responsible for t(14;18) formation (23,28). We found that t(14;18) breakpoints in the BCL-2 major breakpoint region (MBR) on chromosome 18 cluster around positions 3520, 3571 and 3629 on NM_000633.2, consistent with prior reports on FL cases and healthy individuals (17,29). This quantitative genomic map of t(14;18) breakpoints represents a baseline mutational landscape in these study subjects, and a time-course of such measurements could reveal clonal expansion on the path to lymphoma. For complete details for all t(14;18) clones, including the *de novo* ‘N sequence’ inserts, please see Supplementary Table S1.

## DISCUSSION

Droplet-based dPCR provides a powerful tool for sensitive genetic analysis and has been reported for quantitative detection of point mutations with detection limits of 1 mutant allele in 10^4^–10^5^ wild-type copies (8,9,30). Here, we demonstrated a nested microfluidic dPCR that enables highly sensitive and quantitative detection of rare somatic translocation targets in a vast wild-type DNA background. The hemi-nested primer design reduces non-specific PCR amplification; however, we found that significant improvement in sensitivity is mainly conferred by using preamp reactions to increase the amount of target sequence relative to the overall concentration of gDNA used in the dPCR assay and thus decrease the interference from excessive background. We tested gDNA NTCs [preamp of 3 µg of purely t(14,18)^−^ gDNA] in dPCR and found that the percentage of false FAM^+^ beads was strongly dependent on the average concentration of gDNA in droplets. Excessive background (>0.1% false FAM^+^ beads) was observed when averaged concentrations were >0.1 genomic copies per droplets (∼100 pg/µl), owing to non-specific amplification of concentrated background gDNA in 2.5 nl of droplets. Therefore, it is necessary to operate at 0.01–0.1 copies per droplet to achieve highly sensitive detection. This operational limit results in a large dead volume during droplet generation and lowers the effective throughput of the dPCR assay when used to assay target mutations directly. Preamp of the low concentration standards allows us to use diluted gDNA concentration in dPCR, which enable quantitative detection down to concentrations of 1 × 10^−7^ or even lower (Figure 2b and inset) while maintaining the analysis efficiency.

The microfluidic approach described here has digital detection capability for highly quantitative measurement of low-level t(14;18) mutations with single-molecule sensitivity and resolution. Conventional qPCR assays developed here and by other researchers also conferred single-molecule sensitivity to detect t(14;18) at 10^−5^ to 10^−7^ levels, depending on the amount of gDNA screened (10,16,19,20,31–35). However, these analog measurements remained semi-quantitative, especially at the low concentration range (e.g. <10^−5^ in Figure 2a). Another distinct advantage that the dPCR method offers over analog qPCR assays is that it enables high-throughput targeted single-molecule sequencing and allows various t(14;18) clones to be resolved and quantified individually (Figures 4 and 5). We demonstrated that the microfluidic nested dPCR method provides single-molecule resolution and enables identification, quantitation and genomic mapping of unique t(14;18) clonal forms that are unresolvable using the conventional nested qPCR approach (see data for subjects G, Q, H and HH in Figure 3a, Supplementary Table S1 and Supplementary Figures S4 and S5). This technology thus could provide a powerful tool for investigating the clonal evolution of cancer at the single copy level. Clinical studies have identified clonal evolution of t(14;18) in individual patients in response to disease progression (23). A high-throughput technique with the capability to sequence and quantify multiple t(14;18)^+^ clones could provide insights into the molecular pathology and clinical implication of t(14;18) (24,25). It could also be used to detect rare cancer stem cells in a large background of normal tissue. We may have identified t(14;18)^+^ lymphoma stem cells in this study population, but only continued monitoring in a large prospective study can reveal whether particular t(14;18)^+^ clones expand and give rise to lymphoma. In addition, in a previous report, we have demonstrated that this bead-based dPCR technology can be adapted for multiplexed detection of multiple mutations in single cells, which can provide even deeper insight into disease progression (36).

Furthermore, we demonstrated that our nested PCR assays for t(14;18) can be used directly on gDNA from whole blood or clot, a biological specimen that is easily collected in population-based studies. With few exceptions, researchers assay for t(14;18) in hematopoietic cell subpopulations that are enriched for B cells. We found significant differences in the t(14;18) qPCR signal provided by donor-matched PBMCs, buffy coat and clot, with clot gDNA containing the lowest t(14;18) concentrations (Supplementary Figure S1). However, collection strategies in field-based studies of healthy populations often cannot accommodate immediate blood fractionation, and a high sensitivity and throughput t(14;18) assay using clot gDNA of healthy individuals, such as the method described here, would be useful in large prospective cohort studies.

To our knowledge, this is the first time that a highly sensitive t(14;18) assay has been applied to a healthy Chinese population; though our group previously applied a less sensitive assay to PBMC DNA in a smaller Chinese population (37). This is also the first study to apply microfluidic dPCR to monitor somatic cancer mutations in occupationally exposed human subjects. Through the use of the new microfluidic nested dPCR technology, we detected, sequenced and quantified 60 t(14;18) clones found in 41 of 93 (∼44%) healthy Chinese subjects. Our quantitative genomic mapping of these t(14;18) clones revealed clustering within the MBR region of *BCL2* and within the *IgHJ* locus of chromosome 14, and the clustering we observed is consistent with previous reports. Specifically, we observed clustering within the MBR centered around positions 3520, 3571 and 3629 and mainly involving the J4, J5 and J6 RSSs on the *IgHJ* locus—positions that are essentially identical to those identified in prior reports in Western and North American populations (17,29,38).

The t(14;18) translocation is thought to be an initiating event in FL, and additional mutations after t(14;18) can be associated with various outcome measures (17,24). A study that used competitive genomic hybridization in biopsies from t(14;18)^+^ FL cases showed that gain of chromosome X in males and gains involving chromosomes 2, 3q and 5 were among copy number alterations associated with poor outcome (39). Gene disruptions that are frequently associated with adverse outcome in FL cases include *TNFRSF14* on 1p36 along with *FAS* and *TP53* on 10q and 17p, respectively (40). A cytogenetic study of t(14;18)^+^ FL biopsies found that del(6q), +5, +19 and +20 were associated with poorer overall survival, and that del(17p) was associated with poorer event-free survival (25). Although cytogenetics and FISH offer information about mutation concurrence in single cells, these methods are laborious, low-throughput and do not provide sequence information. Most other modern methods use homogenized samples, and there is no opportunity to achieve large-scale studies of mutation concurrence and synergy during disease progression at the single cell level-where carcinogenesis ultimately occurs.

The dPCR and single-molecule sequencing technology established here provides a promising platform for developing new approaches for high-throughput single-cells analysis of the concurrence of multiple mutations. Based on this digital microfluidic platform, we recently developed a methodology for high-throughput purification of single-cell genomes and multiple-allele sequencing of single cells (36). Currently, the throughput of our single-molecule/cell sequencing procedure is limited by the use of second-round PCR to amplify the bead-bound DNA for the standard Sanger sequencing. However, it is feasible to adapt our bead-based dPCR method to next-generation sequencing technologies for direct massively parallel sequencing off the post-PCR beads, thus providing unprecedented throughput in single-cell genetic analysis. We hope to soon extend this microfluidic single cell analysis technology to an expanded set of genetic markers of lymphoma and conduct multiple allele sequencing of these regions at the single-cell level in lymphoma biopsies. With these further developments, we will add additional dimensions to the mutational landscape developed here so that we can begin to study FL progression at the level of individual cancer stem cells.

## SUPPLEMENTARY DATA

Supplementary Data are available at NAR Online, including [26,41,42].

## FUNDING

Trans-National Institutes of Health Genes, Environment and Health Initiative, Biological Response Indicators of Environmental Systems Center Grant [U54 ES016115-01 to M.T.S. and R.A.M.] and National Institute of Environmental Health Sciences Superfund Basic Research Program Grant [P42 ES004705 to M.T.S.]; Canary Foundation and ACS Postdoctoral Fellowship Award in Early Detection [116373-PFTED-08-251-01-SIED to J.S.] from the American Cancer Society; New faculty start-up funds from the University of Kansas (in part to Y.Z.). National Science Foundation Graduate Research Fellowship (to R.N.). Funding for open access charge: National Institutes of Health [U54 ES016115-01].

*Conflict of interest statement*. None declared.

## Supplementary Material

Supplementary Data

## References

[1] Carter H, Chen S, Isik L, Tyekucheva S, Velculescu VE, Kinzler KW, Vogelstein B, Karchin R (2009). Cancer-specific high-throughput annotation of somatic mutations: computational prediction of driver missense mutations. Cancer Res..

[2] Chiu BCH, Dave BJ, Blair A, Gapstur SM, Zahm SH, Weisenburger DD (2006). Agricultural pesticide use and risk of t(14;18)-defined subtypes of non-Hodgkin lymphoma. Blood.

[3] Maheswaran S, Sequist LV, Nagrath S, Ulkus L, Brannigan B, Collura CV, Inserra E, Diederichs S, Iafrate AJ, Bell DW (2008). Detection of mutations in EGFR in circulating lung-cancer cells. N. Engl. J. Med..

[4] Bene MC, Kaeda JS (2009). How and why minimal residual disease studies are necessary in leukemia: a review from WP10 and WP12 of the European LeukaemiaNet. Haematologica.

[5] Shuga J, Zeng Y, Novak R, Mathies RA, Hainaut P, Smith MT (2010). Selected technologies for measuring acquired genetic damage in humans. Environ. Mol. Mutagen..

[6] Dressman D, Yan H, Traverso G, Kinzler KW, Vogelstein B (2003). Transforming single DNA molecules into fluorescent magnetic particles for detection and enumeration of genetic variations. Proc. Natl Acad. Sci. USA.

[7] Diehl F, Schmidt K, Choti MA, Romans K, Goodman S, Li M, Thornton K, Agrawal N, Sokoll L, Szabo SA (2008). Circulating mutant DNA to assess tumor dynamics. Nat. Med..

[8] Hindson BJ, Ness KD, Masquelier DA, Belgrader P, Heredia NJ, Makarewicz AJ, Bright IJ, Lucero MY, Hiddessen AL, Legler TC (2011). High-throughput droplet digital PCR system for absolute quantitation of DNA copy number. Anal. Chem..

[9] Pekin D, Skhiri Y, Baret JC, Le Corre D, Mazutis L, Salem CB, Millot F, El Harrak A, Hutchison JB, Larson JW (2011). Quantitative and sensitive detection of rare mutations using droplet-based microfluidics. Lab Chip.

[10] Schuler F, Dolken L, Hirt C, Kiefer T, Berg T, Fusch G, Weitmann K, Hoffmann W, Fusch C, Janz S (2009). Prevalence and frequency of circulating t(14;18)-MBR translocation carrying cells in healthy individuals. Int. J. Cancer.

[11] Aster JC, Longtine JA (2002). Detection of BCL2 rearrangements in follicular lymphoma. Am. J. Pathol..

[12] Weiss LM, Warnke RA, Sklar J, Cleary ML (1987). Molecular analysis of the t(14;18) chromosomal translocation in malignant lymphomas. N. Engl. J. Med..

[13] Bakhshi A, Jensen JP, Goldman P, Wright JJ, McBride OW, Epstein AL, Korsmeyer SJ (1985). Cloning the chromosomal breakpoint of t(14;18) human lymphomas: clustering around JH on chromosome 14 and near a transcriptional unit on 18. Cell.

[14] Cleary ML, Sklar J (1985). Nucleotide sequence of a t(14;18) chromosomal breakpoint in follicular lymphoma and demonstration of a breakpoint-cluster region near a transcriptionally active locus on chromosome 18. Proc. Natl Acad. Sci. USA.

[15] Korsmeyer SJ (1999). BCL-2 gene family and the regulation of programmed cell death. Cancer Res..

[16] Rabkin CS, Hirt C, Janz S, Dolken G (2008). t(14;18) Translocations and risk of follicular lymphoma. J. Natl Cancer Inst. Monogr..

[17] Biagi JJ, Seymour JF (2002). Insights into the molecular pathogenesis of follicular lymphoma arising from analysis of geographic variation. Blood.

[18] Bretherick KL, Bu R, Gascoyne RD, Connors JM, Spinelli JJ, Brooks-Wilson AR (2010). Elevated circulating t(14;18) translocation levels prior to diagnosis of follicular lymphoma. Blood.

[19] Agopian J, Navarro JM, Gac AC, Lecluse Y, Briand M, Grenot P, Gauduchon P, Ruminy P, Lebailly P, Nadel B (2009). Agricultural pesticide exposure and the molecular connection to lymphomagenesis. J. Exp. Med..

[20] Roulland S, Navarro JM, Grenot P, Milili M, Agopian J, Montpellier B, Gauduchon P, Lebailly P, Schiff C, Nadel B (2006). Follicular lymphoma-like B cells in healthy individuals: a novel intermediate step in early lymphomagenesis. J. Exp. Med..

[21] Fuscoe JC, Setzer RW, Collard DD, Moore MM (1996). Quantification of t(14;18) in the lymphocytes of healthy adult humans as a possible biomarker for environmental exposures to carcinogens. Carcinogenesis.

[22] Paltiel O, Zelenetz A, Sverdlin I, Gordon L, Ben-Yehuda D (2000). Translocation t(14;18) in healthy individuals: preliminary study of its association with family history and agricultural exposure. Ann. Oncol..

[23] Tsujimoto Y, Gorham J, Cossman J, Jaffe E, Croce CM (1985). The t(14;18) chromosome translocations involved in B-cell neoplasms result from mistakes in VDJ joining. Science.

[24] Bende RJ, Smit LA, van Noesel CJ (2007). Molecular pathways in follicular lymphoma. Leukemia.

[25] d'Amore F, Chan E, Iqbal J, Geng H, Young K, Xiao L, Hess MM, Sanger WG, Smith L, Wiuf C (2008). Clonal evolution in t(14;18)-positive follicular lymphoma, evidence for multiple common pathways, and frequent parallel clonal evolution. Clin. Cancer Res..

[26] Zeng Y, Novak R, Shuga J, Smith MT, Mathies RA (2010). High-performance single cell genetic analysis using microfluidic emulsion generator arrays. Anal. Chem..

[27] Vogelstein B, Kinzler KW (1999). Digital PCR. Proc. Natl Acad. Sci. USA.

[28] Schlissel MS, Kaffer CR, Curry JD (2006). Leukemia and lymphoma: a cost of doing business for adaptive immunity. Genes Dev..

[29] Raghavan SC, Swanson PC, Wu X, Hsieh CL, Lieber MR (2004). A non-B-DNA structure at the Bcl-2 major breakpoint region is cleaved by the RAG complex. Nature.

[30] Taniguchi K, Uchida J, Nishino K, Kumagai T, Okuyama T, Okami J, Higashiyama M, Kodama K, Imamura F, Kato K (2011). Quantitative detection of EGFR mutations in circulating tumor DNA derived from lung adenocarcinomas. Clin. Cancer Res..

[31] Crescenzi M, Seto M, Herzig GP, Weiss PD, Griffith RC, Korsmeyer SJ (1988). Thermostable DNA polymerase chain amplification of t(14;18) chromosome breakpoints and detection of minimal residual disease. Proc. Natl Acad. Sci. USA.

[32] Dolken G, Dolken L, Hirt C, Fusch C, Rabkin CS, Schuler F (2008). Age-dependent prevalence and frequency of circulating t(14;18)-positive cells in the peripheral blood of healthy individuals. J. Natl Cancer Inst. Monogr..

[33] Gribben JG, Freedman A, Woo SD, Blake K, Shu RS, Freeman G, Longtine JA, Pinkus GS, Nadler LM (1991). All advanced stage non-Hodgkin's lymphomas with a polymerase chain reaction amplifiable breakpoint of bcl-2 have residual cells containing the bcl-2 rearrangement at evaluation and after treatment. Blood.

[34] Liu Y, Hernandez AM, Shibata D, Cortopassi GA (1994). BCL2 translocation frequency rises with age in humans. Proc. Natl Acad. Sci. USA.

[35] Rauzy O, Galoin S, Chale JJ, Adoue D, Albarede JL, Delsol G, al Saati T (1998). Detection of t(14;18) carrying cells in bone marrow and peripheral blood from patients affected by non-lymphoid diseases. Mol. Pathol..

[36] Novak R, Zeng Y, Shuga J, Venugopalan G, Fletcher DA, Smith MT, Mathies RA (2011). Single-cell multiplex gene detection and sequencing with microfluidically generated agarose emulsions. Angew Chem. Int. Ed. Engl..

[37] McHale CM, Lan Q, Corso C, Li G, Zhang L, Vermeulen R, Curry JD, Shen M, Turakulov R, Higuchi R (2008). Chromosome translocations in workers exposed to benzene. J. Natl Cancer Inst. Monogr..

[38] Wyatt RT, Rudders RA, Zelenetz A, Delellis RA, Krontiris TG (1992). BCL2 oncogene translocation is mediated by a chi-like consensus. J. Exp. Med..

[39] Eide MB, Liestol K, Lingjaerde OC, Hystad ME, Kresse SH, Meza-Zepeda L, Myklebost O, Troen G, Aamot HV, Holte H (2010). Genomic alterations reveal potential for higher grade transformation in follicular lymphoma and confirm parallel evolution of tumor cell clones. Blood.

[40] Wrench D, Montoto S, Fitzgibbon J (2010). Molecular signatures in the diagnosis and management of follicular lymphoma. Curr. Opin. Hematol..

[41] Zhang L, Tang X, Rothman N, Vermeulen R, Ji Z, Shen M, Qiu C, Guo W, Liu S, Reiss B (2010). Occupational exposure to formaldehyde, hematotoxicity, and leukemia-specific chromosome changes in cultured myeloid progenitor cells. Cancer Epidemiol. Biomarkers Prev..

[42] Kumaresan P, Yang CJ, Cronier SA, Blazej RG, Mathies RA (2008). High-throughput single copy DNA amplification and cell analysis in engineered nanoliter droplets. Anal. Chem..

