# Supplementary material for: Single molecule quantitation and sequencing of rare translocations using microfluidic nested digital PCR
Source: Nucleic Acids Res. 2013 Jul 19;41(16):e159. doi: 10.1093/nar/gkt613 (PMC3763562; doi:10.1093/nar/gkt613)
Supplement: Supplementary Data [file supp_41_16_e159__index.html]

Single molecule quantitation and sequencing of rare translocations using microfluidic nested digital PCR — Single molecule quantitation and sequencing of rare translocations using microfluidic nested digital PCR — Supplementary Data 

# Single molecule quantitation and sequencing of rare translocations using microfluidic nested digital PCR

## 

files

**Files in this Data Supplement:**

- Supplementary Data - doc file
