# Supplementary material for: Single molecule quantitation and sequencing of rare translocations using microfluidic nested digital PCR
Source: Nucleic Acids Res. 2013 Jul 19;41(16):e159. doi: 10.1093/nar/gkt613 (PMC3763562; doi:10.1093/nar/gkt613)
Supplement: Supplementary Data [file supp_gkt613_nar-00657-met-k-2013-File002.doc]

**Single Molecule Quantitation and Sequencing of Rare Translocations Using Microfluidic Nested Digital PCR**

Joe Shuga^1,8^, Yong Zeng^2,3,8^, Richard Novak^4^, Qing Lan^5^, Xiaojiang Tang^6^, Nathaniel Rothman^5^, Roel Vermeulen^7^, Laiyu Li^6^, Alan Hubbard^1^, Luoping Zhang^1^, Richard A. Mathies^2,4^, Martyn T. Smith^1*^,

^1^ School of Public Health, University of California, Berkeley, CA 94720, USA

^2^ Department of Chemistry, University of California, Berkeley, CA 94720, USA

^3^ Department of Chemistry, University of Kansas, Lawrence, KS 66045, USA

^4^ UC San Francisco/UC Berkeley Graduate Program in Bioengineering; University of California, Berkeley, CA 94702, USA

^5^ Division of Cancer Epidemiology and Genetics, National Cancer Institute, NIH, Department of Health and Human Services, Bethesda, Maryland, USA

^6^ Guangdong Poisoning Control Center, Guangzhou, PR China;

^7^ Environmental Epidemiology Division, Institute for Risk Assessment Sciences, Utrecht University, Utrecht, the Netherlands

^8^ The authors wish it to be known that, in their opinion, the first two authors should be regarded as joint First Authors.

* To whom correspondence should be addressed. Tel: 1-510-642-8770; Fax: 1-510-642-0427; Email: martynts@berkeley.edu

**Supplementary methods**

Study Subjects: The study population and exposure assessment have been previously described in detail (41). This study was approved by the Institutional Review Boards at the U.S. National Cancer Institute and the Guangdong Poisoning Control Center. Participation was voluntary and written informed consent was obtained. A formaldehyde-exposed worker population was enrolled from two factories, one that produced a formaldehyde-melamine resin, and one that used formaldehyde-melamine resin to manufacture plastic utensils. A control population was selected from three workplaces in the same geographic region as the factories with formaldehyde exposure, and this control population consisted of workers with similar demographic and socioeconomic characteristics and who were primarily engaged in manufacturing. All exposed workers met two inclusion criteria: (1) they had formaldehyde exposure levels of ~1-2 ppm on most days during the initial screening; and (2) they held the same job for at least the previous three months in the same factory. Exclusion criteria for both exposed and control populations were history of cancer, chemotherapy and radiotherapy, as well as previous occupations with notable exposure to benzene, butadiene, styrene, and/or ionizing radiation. We enrolled 43 study subjects exposed to relatively high levels of formaldehyde (mostly between 0.6 and 2.5 ppm), and we enrolled 51 controls who were frequency matched by age (+/- 5 years) and gender to the exposed workers. For more detail regarding the demographic characteristics of the study subjects, please see Supplementary Tbl. 2. Data on t(14;18) were not available for one of the 43 formaldehyde-exposed workers. In addition to the 93 study subjects that were tested for t(14;18), one of the authors carries low levels of t(14;18) and this individual provided peripheral blood for comparison of relative signal in clot, buffy coat, and PBMCs (Supplementary Fig. 1).

Exposure Assessment: Formaldehyde exposure was monitored with UMEx 100 diffusion samplers, and the limit of detection was 0.012 ppm. These samplers were worn by workers in the exposed workplaces for a full shift (>240 min) on ~3 working days over a 3-wk period. Biological samples were collected toward the end of this period. Each formaldehyde-exposed subject had a minimum of two diffusion samplers collected. The average formaldehyde exposure level was calculated by taking the arithmetic mean of each subject’s measurements. A subgroup of workers in the unexposed workplaces was monitored for formaldehyde exposure on a single day. For more detail regarding the exposure characteristics of the study subjects, please see Supplementary Tbl. 2.

Biological Sampling: In June and July 2006, we obtained biological samples from the 94 study subjects at their workplaces after informed consent was obtained. Biological samples were collected after the formaldehyde-exposed workers had been monitored at least twice for personal formaldehyde exposure in their workplace. Peripheral blood samples were collected from each study subject and delivered to the processing laboratories within 4 hours of collection. Peripheral blood from each study subject was drawn into different vacutainer tubes by specially trained nurses, and was then allowed to clot before shipment to Bioreliance, Inc. under contract to the National Cancer Institute. Bioreliance then extracted clot gDNA, recoded the samples, and sent the samples to the University of California at Berkeley laboratory for analysis. Thus, all laboratory analyses carried out after completion of the field phase of the study were done blinded to exposure status.

Cell and gDNA Purification: For purification of clot gDNA we used Clotspin® baskets and the Gentra® Puregene® blood kit according to the manufacturer’s recommendations (Qiagen, Valencia, CA). Buffy coat was prepared by spinning whole blood at 200x g for 10 min at room temperature and then removing the concentrated leukocyte band plus a small portion of the plasma and concentrated red blood cells. We then isolated DNA from buffy coat using the FlexiGene DNA kit and the manufacturer’s instructions (Qiagen). PBMCs were purified from whole blood using density gradient centrifugation through Ficoll-Paque™ PLUS according to the manufacturer’s recommendations (GE Healthcare, Piscataway, NJ). To purify gDNA from cell lines and PBMCs we used a standard cell lysis with RNA and protein digests followed by a phenol-chloroform DNA extraction (solvents from Sigma-Aldrich, St. Louis, MO). Briefly, cells (5x10^6^/mL) were lysed in a 10 mM Tris-HCl buffer (pH=8.0) with 100 mM EDTA and 0.5% SDS and then treated first with RNAse (20 mg/L, from Roche) at 37 ^o^C for 1 hour and then with Proteinase K (100 mg/L, from Qiagen) at 56 ^o^C for 3-24 hours. Phenol-chloroform extraction was then performed in Phase Lock Gel^TM^ tubes (Eppendorf) using an initial wash with phenol/chlorofrom/isoamyl alcohol (25/24/1, v/v/v, [PCI]), followed by a second Proteinase K treatment (50 mg/L, 2-4 hours at 56 ^o^C), followed by three more washes in PCI, and finally followed by a single wash with chloroform/isoamyl alcohol (24/1, v/v). DNA was then precipitated by adding ammonium acetate to 500 mM and isopropyl alcohol to ~43% (v/v). DNA was spooled onto a pipette tip and washed thrice in 70% ethanol before air drying. DNA was then resuspended in Tris-EDTA buffer (10 mM Tris-HCl, 5 mM EDTA, pH=8.0) and the concentration was measured by UV absorption (A_260_) and adjusted to 200ng/µL. The quality of gDNA was assessed and copy number was normalized using quantitative PCR for β-actin. The β-actin PCR mix contained 1X *Ampli* Taq Gold® buffer with 5 mM MgCl_2_, 0.2 mM dNTPs (dUTP was used at 0.4 mM instead of dTTP), 0.01 U µL^-1^ uracil DNA glycosylase (UDG, Roche), 2.5% DMSO, 1 µM ROX reference dye, 0.3 µM each of the oligonucleotides (F-ActB, R-ActB, ActB probe, please see Supplementary Tbl. 3 for sequences), 0.035 U µL^-1^ *Ampli* Taq Gold® Polymerase and 1 µg gDNA per 50 µL reaction. Thermal cycling in an ABI 7300 cycler consisted of a UDG reaction (50 ^o^C for 2 min), followed by a 10 min hot start at 95 ^o^C, and 40 cycles of 95 ^o^C for 15 s, 60 ^o^C for 30 s, and 72 ^o^C for 30 s.

MEGA Device fabrication and assembly: The 4-channel MEGA devices were constructed from 100-mm diameter glass wafers and a thin poly(dimethylsiloxane) (PDMS) membrane, as detailed previously (26). Briefly, the valve seat structure was first microfabricated on the top side of a Borofloat® glass wafer. Using backside alignment lithography, the microchannels were patterned onto the bottom of the same wafer in register with the pump structure. Fluidic and interlayer access holes were drilled and the wafer was thermally bonded to a clean substrate to enclose the microfluidic network. The on-chip pump assembly was completed by sandwiching a 200 µm PDMS membrane between the chip and a pneumatic manifold plate with microfabricated valve displacement chambers. The glass channels were rendered hydrophobic by coating with a 0.1% solution of octadecyltrichlorosilane (OTS, Sigma-Aldrich) in dry toluene for 10 min.

Bead functionalization: All reagents were handled in a UV-treated laminar flow hood (UVP, Upland, CA). All oligonucleotides used in this study (Supplementary Tbl. 3) were obtained from IDT (Coralville, IA). For emulsion dPCR, the Nv3 primer was labeled with 6-FAM on the 5' end. We added a C_12_ spacer and a 5' amine modification to JH Exo and then covalently linked the primer to 6% cross-linked NHS-activated Sepharose beads (34 μm mean diameter, #17-0716-01, GE Healthcare) at ~0.5 µmol g^-1^ through amine-NHS conjugation chemistry (26). After overnight incubation, primer beads were washed and re-suspended in water at final bead concentrations of 4000-8000 beads µL^-1^.

Droplet generation: The fresh carrier oil was prepared daily and contains 39.8% (w/w) DC 5225C Formulation Aid (Dow Chemical Co., Midland, MI), 30% (w/w) DC 749 Fluid (Dow Chemical Co.), 30% (w/w) AR20 Silicone Oil (Sigma-Aldrich), and 0.2% (w/w) Triton X-100 surfactant (Sigma-Aldrich). Following the completion of this work, we have discovered that recent batches of 749 Fluid exhibit decreased emulsion stability.  We find that omitting Tween 80 from the PCR cocktail is sufficient to restore stability during thermal cycling. The MEGA device was assembled with a custom aluminum manifold which provides fluidic connection for oil infusion and droplet collection. Carrier oil was continuously injected into the MEGA device from two gastight syringes (Hamilton Company, Reno, NV) using a syringe pump (PHD 2000, Harvard Apparatus, Holliston, MA). Emulsion dPCR mix containing t(14;18) amplicon and primer beads was injected into the microchannels for droplet generation by the on-chip 3-valve diaphragm pump, which was pneumatically actuated by a solenoid valve controller system built in house. The pumping was conducted in a four-step fashion under the control of a LABVIEW graphical interface (National Instruments, Austin, TX) ([42](#_ENREF_1)).

Pumping parameters were optimized to produce uniform ~2.5 nL PCR reaction droplets (actuation times: valves 1 and 2 open, 42 ms; valve 1 close, 42 ms; valve 3 open, 20 ms; valves 2 and 3 close, 18 ms; total oil flow rate: 14 µL min^-1^; pneumatic line pressure/vacuum: +35/-85 kPa). The sample reservoir was constantly replenished with PCR mix to sustain the droplet generation. PCR droplets were collected in 0.5 mL PCR tubes (Eppendorf) filled with microfine emulsion.

Digital PCR: The dPCR mix contained 1x *Ampli* Taq Gold® buffer with 3 mM MgCl_2_, 0.2 mM dNTPs, 0.4 µg/µL heat-inactivated BSA, 0.01% Tween 80, 0.3 µM FAM-Nv3, 0.03 µM JH Exo, 0.125 U µL^-1^ *Ampli* Taq Gold® Polymerase, ~85 beads functionalized with JH Exo per µL (target ~0.2 beads per 2.5-nL droplet), and 0.5-0.05 µL of preamp product per 50 µL dPCR reaction volume (titrated to achieve 0.01-0.2 copies per 2.5-nL droplet). For preamp reactions with low t(14;18) frequencies, 0.5 µL of the preamp reaction yielded droplets that were sufficiently dilute to quantify t(14;18) (i.e. not saturated). However, for some of the higher t(14;18) concentration reactions, 0.5 µL of the preamp reaction yielded droplets that were 100% t(14;18)^+^. These high t(14;18) concentration samples were diluted 10-fold in water and reanalyzed (i.e. 0.05 µL of product from the preamp reaction was used per 50 µL dPCR reaction volume for these high concentration samples). Thermal cycling was carried out in a PTC200 thermocycler (MJ Research, Waltham, MA) and involved a 10 min hot start at 95 ^o^C, and 33 cycles of 95 ^o^C for 30 s, 60 ^o^C for 60 s, 72 ^o^C for 90 s, and a final 72 ^o^C extension for 5 min. The reactions were then cooled to 4 ^o^C until bead recovery (typically overnight).

Bead recovery and flow cytometry quantitation: The dPCR reaction tube was vacuumed to pass through a 15 μm mesh filter (Qosina, Edgewood, NY) held in a 13 mm-diameter plastic filter holder (Pall Life Sciences, Ann Arbor, MI). The droplets were broken and the beads were retained by the filter. After rinsing with isopropanol, ethanol and 1× Dulbecco’s PBS (DPBS, GIBCO) (~15 mL each), the beads were recovered using a 5 mL syringe and stored in DPBS for flow cytometry analysis. The bead suspension in DPBS was analyzed by using a multicolor flow cytometer (FC-500, Beckman-Coulter, Fullerton, CA). The flow cytometry data were analyzed by using WinMDI (Scripps, La Jolla, CA) and WinList 6.0 (Verity Software, Topsham, ME).

Techniques to prevent PCR contamination: In addition to using UDG in preamplification, we also used dedicated work spaces for pre- and post-PCR work with filter pipette tips. All components of PCR mixes were prepared in a sterile laminar-flow hood; and the sterile hood and the pipettes used to set up PCR reactions underwent routine UV sterilization between experiments. In addition, all samples that were positive based on cleavage of the probe sequence in qPCR, or based on concentration of FAM^+^ beads in dPCR, were then confirmed by sequencing the clonal form of t(14;18). In the rare event that the t(14;18) sequence from the CRL 2261 positive control cell line (3615-CGGCCCTTTAGGATCCC-87330523 [BCL2-N-IgHJ]) was identified, the “positive” result of that particular assay was disqualified. Negative controls (from preamp of CRL 8015 gDNA) were included on every nested qPCR assay 96-well plate, and a negative control sample was also included in every run through the droplet generator, and in all cases these negative control reactions tested negative for t(14;18).

Single-molecule sequencing: Single molecule sequencing was accomplished by re-amplifying amplicon-bound beads from dPCR in 50 µL t(14;18) qPCR reactions in 96 well PCR plates. PCR mix contained 1X *Ampli* Taq Gold® buffer with 5 mM MgCl_2_, 0.2 mM dNTPs (dUTP was used at 0.4 mM instead of dTTP), 2.5% DMSO, 1 µM ROX reference dye, 0.3 µM each of the nucleotide sequences (JH Exo, Nv3, and BCL2MBRTM2), and 0.035 U µL^-1^ *Ampli* Taq Gold® Polymerase. Beads were counted using a hemocytometer and added at ~1 bead per 50 µL reaction. Thermal cycling in an ABI 7300 cycler consisted of a 10 min hot start at 95 ^o^C, and 20 cycles of 95 ^o^C for 15 s, 60 ^o^C for 30 s, and 72 ^o^C for 30 s. The bead-bound amplicon was very stable and could be re-amplified even after six months of storage at 4 ^o^C. Reaction products were loaded on a 1.5% agarose gel and electrophoresed to separate various amplicons that were then excised, purified using a QIAquick Gel Extraction Kit (QIAGEN), and sequenced on an ABI 3730 at the UC Berkeley Core Sequencing Facility.

Sequence Analysis: Sequencing reads were aligned to the reference assembly of the human genome using NCBI’s nucleotide basic local alignment search tool (BLASTn at www.ncbi.nlm.nih.gov/blast/). The chromosome 14 breakpoint was defined as the terminus of the best alignment with NT_026437.11 and the chromosome 18 breakpoint was defined as the terminus of the best alignment with NM_000633.2. The “N sequence” insert was identified as the *de novo* sequence found between the two breakpoints in a particular translocation clone (see Fig. 4 for a graphical representation of the sequence analysis). Typically, a single sequencing read of amplicon generated with the Nv3 primer provided sufficient length and quality to fully define the particular clonal form(s) of t(14;18) in a given PCR reaction. However, in some cases, the chromosome 18 breakpoint fell too close to the Nv3 primer location, and it was then necessary to generate a paired read using the JH Exo primer. These paired reads were then aligned to each other to generate a full amplicon-length consensus read that spanned completely from Nv3 to JH Exo. This consensus read was then aligned to the human genome using BLASTn to define the breakpoints on chromosomes 14 and 18 as well as the N sequence insert.

Finally, to map the locations of the V(D)J recombination signal sequences (RSSs) to the chromosome 14 contig (NT_026437.11), we mapped all allowable RSS nonamers and heptamers to the contig and then found nonamer-heptamer pairs that were separated by appropriately sized spacer sequences (please see Fig. 5 and Supplementary Fig. 6).

Statistical Analysis: Quantitative analysis and linear regression of t(14;18) concentrations for subjects and for specific clonal forms were performed in Excel (Microsoft, Redmond, WA). Data is presented as average +/- standard deviation for replicate measurements, except where otherwise indicated. We quantified the amount of a t(14;18) clonal form using the overall t(14;18) concentration from hemi-nested PCR weighted by the relative amount of a clonal form as determined by dPCR.

**References**

26. Zeng, Y., Novak, R., Shuga, J., Smith, M.T. & Mathies, R.A. High-performance single cell genetic analysis using microfluidic emulsion generator arrays. *Anal Chem* 82, 3183-3190 (2010).

41. Zhang, L. et al. Occupational exposure to formaldehyde, hematotoxicity, and leukemia-specific chromosome changes in cultured myeloid progenitor cells. *Cancer Epidemiol Biomarkers Prev* 19, 80-88 (2010).

42. Kumaresan, P., Yang, C.J., Cronier, S.A., Blazej, R.G. & Mathies, R.A. High-throughput single copy DNA amplification and cell analysis in engineered nanoliter droplets. *Anal Chem* 80, 3522-3529 (2008).

**
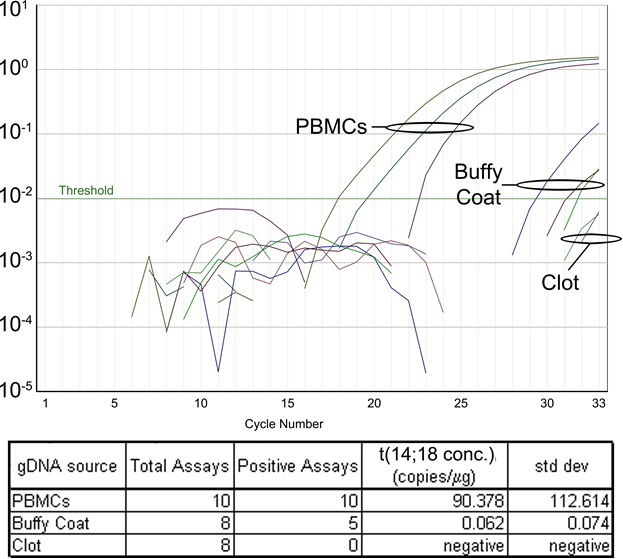
**

**Supplementary Figure 1.**

Comparison of t(14;18) concentration in the matched gDNA from various blood fractions. Peripheral blood was collected from a single t(14;18)+ subject and was split for processing in three different manners prior to isolation of gDNA: (1) whole blood was allowed to clot, (2) whole blood was centrifuged to obtain buffy coat (BC), and (3) whole blood was centrifuged in Ficoll-Paque™ to obtain peripheral blood mononuclear cells (PBMCs). These various blood fractions were then assayed for t(14;18) concentration using qPCR. The amplification curves (delta RN vs. cycle number) are shown for a typical experiment where each blood faction was assayed in triplicate. The PBMC and BC gDNAs undergo sufficient amplification to cross threshold in this trial, but no assays of clot gDNA cross threshold (though 2/3 clot gDNA reactions show some indication of amplification very late in the PCR). The overall results from several different experiments are then summarized in the table below the amplification curves. The t(14;18) concentration in the PBMC gDNA of this subject was ~1000-fold higher than the signal found in the BC gDNA. Furthermore, the t(14;18) concentration in BC was substantially higher than that measured in clot gDNA, with t(14;18) detectable in 5/8 BC assay reactions vs. 0/8 assay reactions on clot gDNA.

**
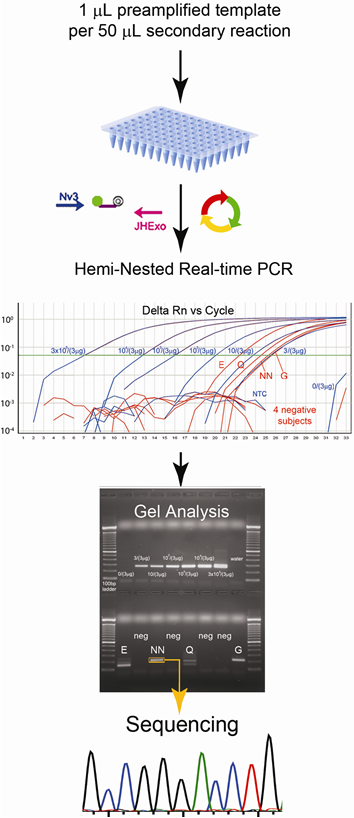
**

**Supplementary Figure 2.**

Standard hemi-nested qPCR. Bulk analysis of template is conducted in 50 μL PCR reactions and a BCL2-specific probe is used to provide a real-time signal for positive reactions. During typical screening, only positive reactions are subjected to gel analysis and sequencing. However, in this illustration of the assay, the results for four positive subjects are shown along with the results for four negative subjects. Following gel separation, amplicon from these reactions is used in sequencing reactions to fully define the clonal form(s) of t(14;18) in a sample.


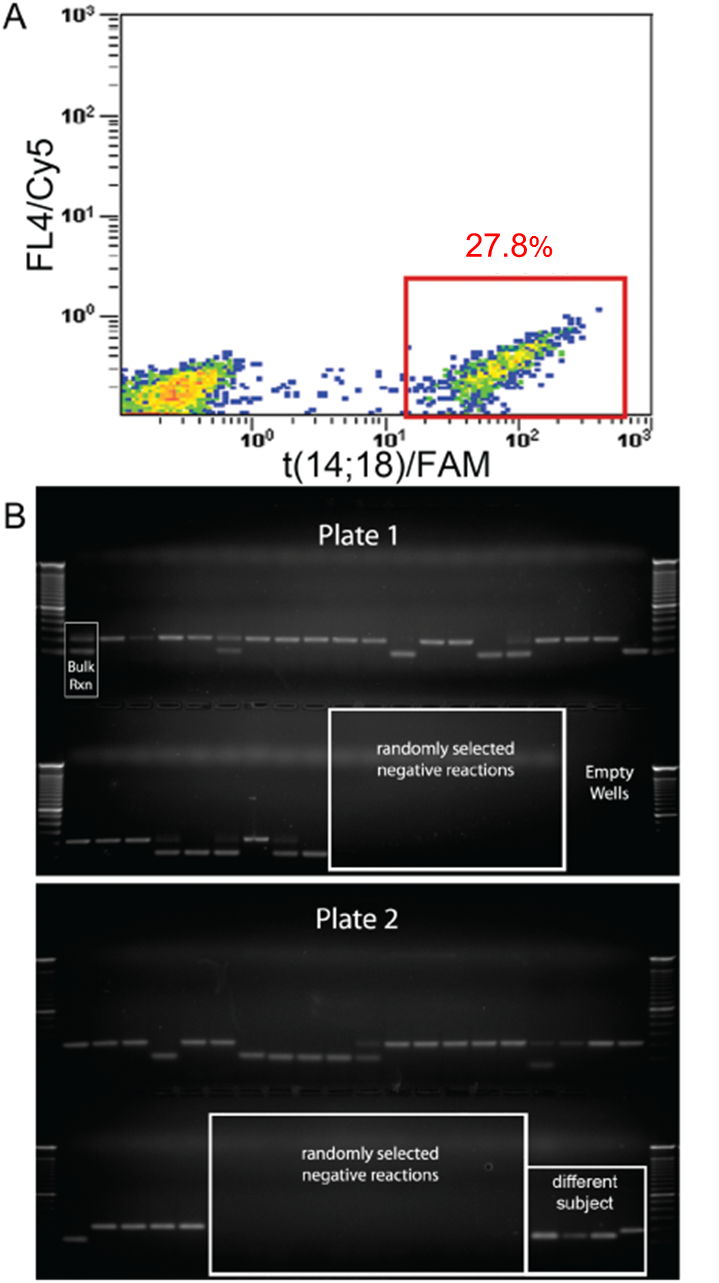
**Supplementary Figure 3.**

Full Gels for Two 96-well plates of dPCR Beads from Subject “D”. (A) Flow cytometry plot showing that the beads from dPCR on this subject are ~28% FAM+. (B) When the beads were plated at ~1 bead per reaction and analyzed by digital real-time PCR, 53 of the 192 wells on two 96-well plates (~27.6% of wells) gave a positive signal from the BCL2-specific probe. When these 53 positive reactions were run on a gel and the clonal forms were counted, it was determined that the ratio of the small form (“clone 1”) to the large form (“clone 2”) was 18:37 in these 53 wells. Two of these 53 bead-templated reactions yielded both clonal forms, indicating either that two positive beads were loaded in a single well on the 96-well plate, or that two copies of t(14;18) were co-encapsulated within a single nL-scale reaction droplet.

**
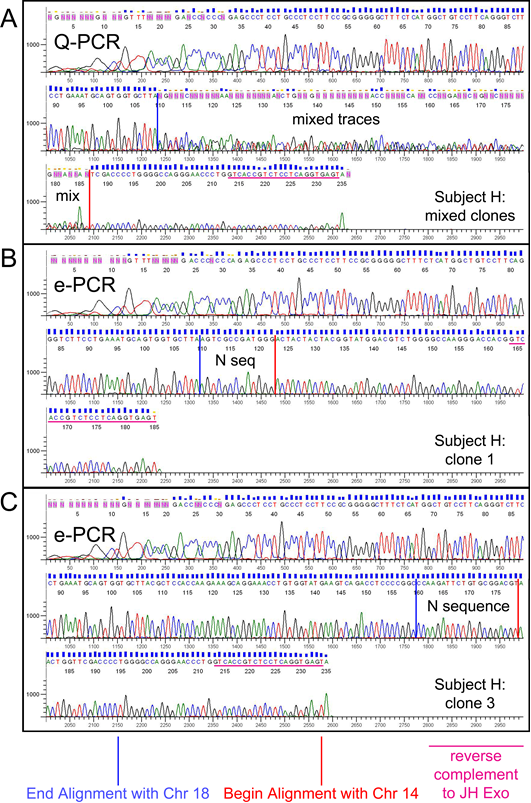
**

**Supplementary Figure 4.**

Resolving Clones of Similar Size in Subject H using dPCR. It was difficult to resolve two of the three clonal forms of t(14;18) that were present in Subject H using standard qPCR and gel analysis. However, microfluidic dPCR was capable of resolving these similarly sized clones. The key features from sequence analysis are indicated on the electropherograms, including: (1) the end of alignment with Chromosome 18; (2) the N sequence (or mixed trace portion); (3) the beginning of alignment with Chromosome 14; and (4) the reverse complement to JH Exo (end of amplicon). (A) A mixed trace primed with Nv3 following standard qPCR and gel analysis. (B) The smaller of the clonal forms (Subject H clone 1, 215 bp) is resolved following dPCR. (C) The larger of the clonal froms (Subject H clone 3, 266 bp) is resolved following dPCR.

**
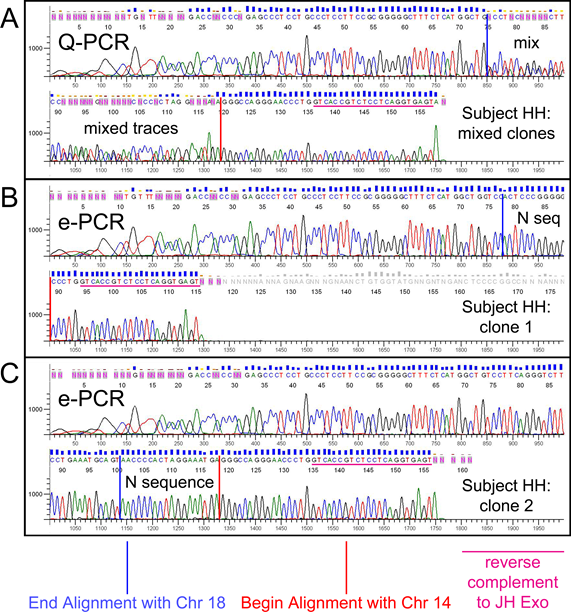
**

**Supplementary Figure 5.**

Resolving Clones of Similar Size in Subject HH using dPCR. It was difficult to resolve the two clonal forms of t(14;18) that were present in Subject HH using standard qPCR and gel analysis. However, microfluidic dPCR was capable of resolving these similarly sized clones. The key features from sequence analysis are indicated on the electropherograms, including: (1) the end of alignment with Chromosome 18; (2) the N sequence (or mixed trace portion); (3) the beginning of alignment with Chromosome 14; and (4) the reverse complement to JH Exo (end of amplicon). (A) A mixed trace primed with Nv3 following standard qPCR and gel analysis. (B) The smaller of the clonal forms (Subject HH clone 1, 145 bp) is resolved following dPCR. (C) The larger of the clonal froms (Subject HH clone 2, 187 bp) is resolved following dPCR.

**
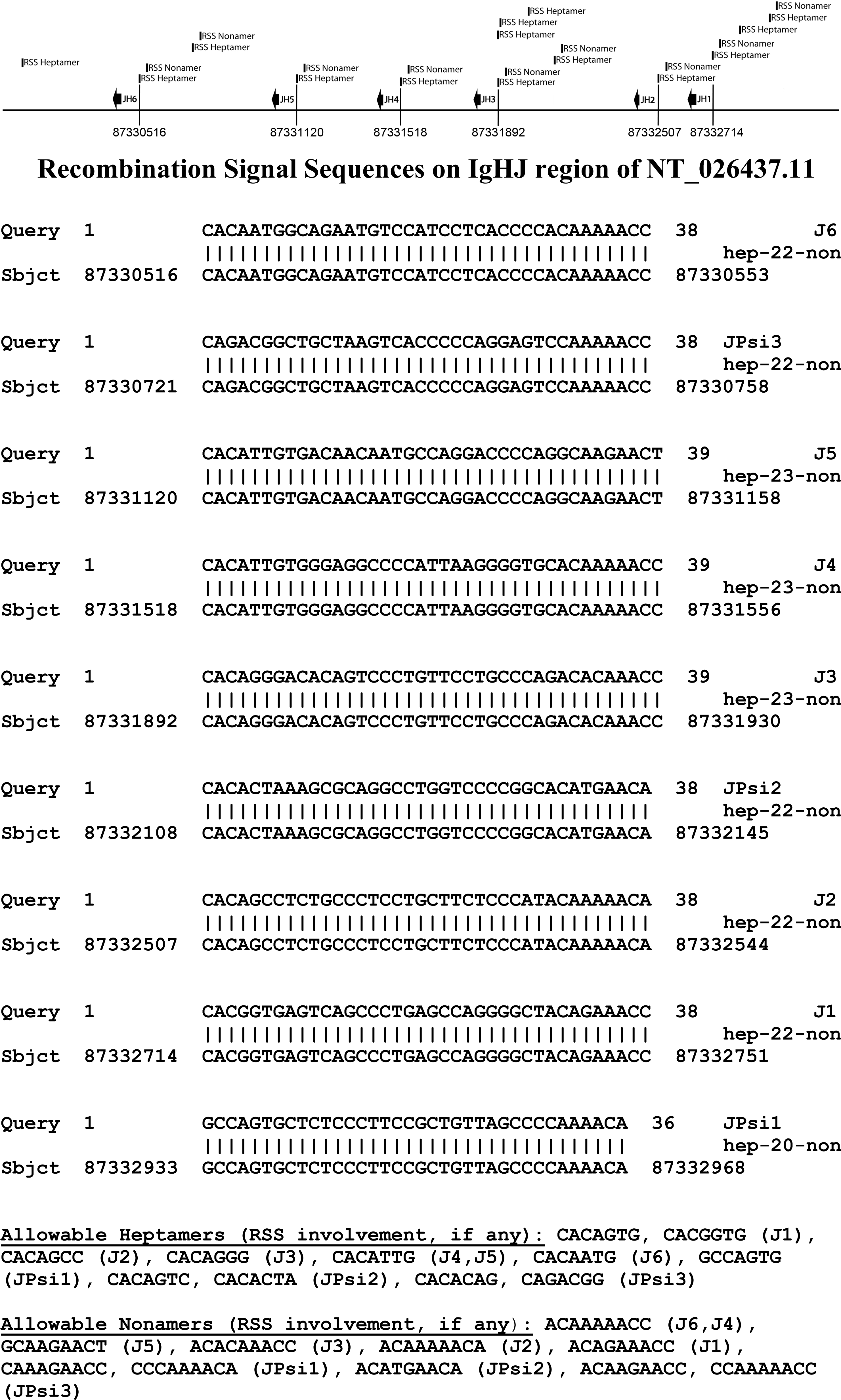
**

**Supplementary Figure 6.** Mapping Recombination Signal Sequences (RSSs) on Chromosome 14 contig NT_026437.11. Map of all RSS heptamers and nonamers on the IgHJ region of Chromosome 14. Appropriately spaced nonamer-heptamer pairs were confirmed as RSSs and mapped to NT_026437.11. If an RSS location corresponded with Chromosome 14 breakpoints observed in this study then the RSS was displayed on Figure 5.

**Supplementary Table 1.** Further Details of t(14;18) Clonal Forms. Breakpoint coordinates are given for all the t(14;18) clones defined in these study subjects. The values in the table refer to position on NT_026437.11 (Chromosome 14 breakpoints) and position on NM_000633.2 (Chromosome 18 breakpoints). The unique “N sequence” insert of these clones is also provided (the N sequence is not shown on Figure 5). The two clonal forms that were discovered only through dPCR are highlighted (G clone 3 and Q clone 3). It should also be noted that the clonal forms for subjects H and HH could not be resolved without the use of emulsion dPCR. Alternative Chromosome 14 alignments, if found, are also given.

**Supplementary Table 2.** Demographic and exposure characteristics of study subjects from Guangdong, China. Modified and reprinted, with permission from (L. Zhang, X. Tang, N. Rothman et al., Cancer Epidemiol Biomarkers Prev 19 (1), 80 (2010)).

**Supplementary Table 3.**

Oligonucleotides used as primers and probes in this study. The sequence, modifications, and uses of the various oligonucleotides in this study.
